# Bayesian inference of spatial and temporal relations in AI patents for EU countries

**DOI:** 10.1007/s11192-023-04699-1

**Published:** 2023-04-29

**Authors:** Krzysztof Rusek, Agnieszka Kleszcz, Albert Cabellos-Aparicio

**Affiliations:** 1 AGH University of Krakow, Kraków, Poland; 2 Jan Kochanowski University of Kielce, Kielce, Poland; 3 Barcelona Neural Networking Center, Universitat Politécnica de Catalunya, Barcelona, Spain

**Keywords:** Artificial intelligence, Patent cooperation network, European Union, A14, C11, C53, O52

## Abstract

In the paper, we propose two models of Artificial Intelligence (AI) patents in European Union (EU) countries addressing spatial and temporal behaviour. In particular, the models can quantitatively describe the interaction between countries or explain the rapidly growing trends in AI patents. For spatial analysis Poisson regression is used to explain collaboration between a pair of countries measured by the number of common patents. Through Bayesian inference, we estimated the strengths of interactions between countries in the EU and the rest of the world. In particular, a significant lack of cooperation has been identified for some pairs of countries. Alternatively, an inhomogeneous Poisson process combined with the logistic curve growth accurately models the temporal behaviour by an accurate trend line. Bayesian analysis in the time domain revealed an upcoming slowdown in patenting intensity.

## Introduction

Cooperation between the member states on issues of common interest was one of the reasons to establish the European Union (EU). The EU is an international organization of FUND contrasting geo-political concepts. On the one hand it resembles a federation, yet on the other the member states have large independence and freedom of foreign cooperation. Patent interactions are a form of cooperation. In this study, we attempt to quantify internal and external EU patent interactions as well as the European trend in the field of Artificial Intelligence (AI). Although there is no internationally agreed definition of AI it could be generally defined as intelligence demonstrated by machines. A more formal definition from (Copeland, 2020) states that it is “the ability of a digital computer or computer-controlled robot to perform tasks commonly associated with intelligent beings”. AI as a concept was first created in the 1950 s but its market relevance has been recognised worldwide in the last decade, mainly due to the development of high-performance parallel computing chips and the availability of large datasets that have extended this technology’s applicability (Leusin et al., 2020).

AI is not a static area of research. Over the past few decades we observed rises and falls of various trends and methods. There are periods of low interest in AI known as AI winter (Schuchmann, 2019). Starting around 2012 we could observe the beginning of a new method known as deep learning that quickly caught the attention of researchers and industry.

Modern AI requires two enablers: data and computational power. While the technology is available to everyone, the groundbreaking work is usually done by big tech companies that have all the required resources and skilled employees at their disposal. Those employees usually come from academia and often work in remote branch offices. This makes the top-tier AI application a group effort involving multiple countries. Such technologies are an important driver of economic growth at national and regional level of competitiveness (Klinger et al., 2018). The technology will significantly contribute to improvements in human welfare across a wide range of sectors, including healthcare, education, transportation, robotics, public safety, employment or entertainment (among others). AI could also lead to groundbreaking discoveries as e.g. predicting protein structure. This development could help supercharge the discovery of new drugs to treat disease, alongside other applications (Senior et al., 2020).

The growth in recent years in AI is a topic of interest in multiple publications e.g. (Cioffi et al., 2020; Goodell et al., 2021; Niu et al., 2016). Having said that we point out that AI can also adversely affect sustainable development but this does not happen frequently. Vinuesa et al. (2020) analysed how AI might impact all aspects of sustainable development in terms of 17 Sustainable Development Goals (SDGs) and its 169 targets internationally agreed in the 2030 Agenda for Sustainable Development. For that study, the authors divided the SDGs into the following three main groups: Society, Economy, and Environment. The results show that 67 targets (i.e., 82%) in the Society main group could potentially benefit from AI-based technologies. In Economy 42 targets benefit from AI (i.e., positive impact in 70% of targets). For the Environment group, results showed that AI could act as an enabler in 93% of targets.

In an increasingly knowledge-driven economy, society invariably needs creative or inventive ideas or concepts to improve existing features and add/develop useful new features to products. All the positive aspects of AI are accessible for the customers as a service or product developed by a company and usually protected by a patent. Also regions, enterprises, and communities that own the most patents are considered more technologically advanced. This brings us to the conclusion that the current landscape of AI patents is a proxy for the future landscape of high-tech industry. In particular we focus on cooperation in patents, as we expect it to be mirrored by the cooperation in products.

Patents are an effective method of measuring the potential for specific technology areas. Their important role in research on innovation is due to their usefulness for examining various technologies in fine detail. A core message from the microanalysis of patents and innovations by Gittelman (2008) is that they are useful indicators of innovation. Furthermore, patents are a long-term investment. The patent applicant can commercialize the invention at any point during that time, either through developing products or services incorporating the patented technology or by licensing it to others. Patent literature and patent information are important sources of information for scientometrics and provide critical guidance for scientific research, business operations and technological innovation (Tsay & Liu, 2020; Wu et al., 2021).

AI has been one of the key drivers of the massive increase in industrial revolution-related patenting over the past decade. Many of the biggest technology companies have invested heavily into AI related research and development. For example, Google, Microsoft, IBM, and Samsung have each submitted thousands of patent applications (Liu, 2020). The scale of AI patents is further confirmed by *OECD.Stat* OECD.Stat (2021) where patent counts are provided for selected technology areas, and technologies related to AI are distinguished as separate technology domains and IPC classes. In general the identification of all the AI patents could be demanding, as the topic is broad and evolves.

In 2019, World Intellectual Property Organization (WIPO) emphasized that despite the availability of information in patent documents, it can be difficult to identify exactly which patent families relate to AI because of the lack of a standardized definition (WIPO, 2019). The literature proposes a variety of strategies for identifying AI-related documents, including the use of predefined classes based on patent classification schemes e.g. (Fujii & Managi, 2018), the use of specific keywords e.g. (Aristodemou & Tietze, 2018; Niu et al., 2016), or even both. Both strategies have pros and cons, and outcomes from their use could bring slightly different results (Leusin et al., 2020). Since the field of AI is dynamically developing the proposed keywords are quickly becoming outdated e.g set of keywords by IPO (2019) is missing many keywords characteristic of contemporary AI technologies e.g. TensorFlow, JAX, generative model or attention model. Thus we relied on predefined patent properties from (IPO, 2019). These are more formalized and detailed thus should better represent the true aspect of the invention. Another important property of each patent is the country of its inventors (one or more). So a patent with multiple countries can be interpreted as a relation between countries.

In a globalized world, international cooperation plays a vital role in tackling global issues including research and science. (Hervás-Oliver et al., 2021) showed a positive effect on SMEs innovation and R &D due to collaboration with others. Similarly the European Commission recognises the importance of global level cooperation to grand societal challenges, hence wants Horizon Europe to open up eligibility to strong science countries (e.g., Canada, Australia, etc.). The problem concerning innovation in Europe was highlighted in the literature. One example is that frequently, the EU-13 (countries that joined the EU in and after 2004) were found at the lower end of participation rankings concerning Horizon 2020. Abbott and Schiermeier (2019) identified that the majority of nations with smaller research budgets are former communist countries in central and eastern Europe, which together with Cyprus and Malta joined the EU after 2004. Further research into the collaboration structure between countries indicated the strongest collaboration research network was between EU-15 (countries that entered the EU before 2004) particularly between Germany, France, United Kingdom, Italy and Spain.

Besides grouping countries, the network of relations can also reveal their strengths as reported by Wu (2020). This particular analysis focuses on collaborations on AI-related papers in journals tracked by the Nature Index. Cooperation based on publications can be biased toward academic interaction. The best universities in Europe are based in United Kingdom and the findings of Wu (2020) are that three United Kingdom institutions are among the biggest collaborators in AI research in Europe. Companies may not be willing to publish their results in scientific journals though they definitely will benefit from patents. We argue that a patent cooperation network is a better tool for the job when it comes to cooperation in the industry. We further argue that analysis of such a cooperation can give a better picture of technology development trends (You et al., 2017).

Patent cooperation is a common business activity and patents are often owned by multiple assignees. The patents that have multiple different assignees are hereafter referred to as *cooperation patents*. The cooperative relationship formed between the assignee is a channel for the transmission of knowledge, information, and resources. The network relationship structure also determines the action opportunities and results of the assignee (Tsay & Liu, 2020).

It is also worth noting that efficient patenting collaboration among different countries could be a challenge because of differential factors between countries, such as geographic distance, economic disparity, language and others highlighted and analysed in the literature [e.g. (Tang et al., 2022; Acosta et al., 2011; Fritsch & Wyrwich, 2018)].

Considering all aforementioned aspects and the importance of the role of patents, it is crucial to analyse cooperation between assignees, especially in the advanced, developing field of AI technologies. It is also worth noting the importance of AI for EU competitiveness is highlighted in many actions i.e., in strategies, regulations, plans, investment and funding programs. Although there are some publications about patent cooperation analysis (including AI patents between assignees) there is a lack of reliable cooperation analysis between EU countries concerning AI patents—including investigation on cooperation between the Member States and the influence of cooperation on patents performance.

The closest related work is by Tsay and Liu (2020) who utilised the Derwent World Patents Index, by using the patent metric and basic graph properties like centrality to investigate the global cooperative network structure of the assignee’s in AI patents. In this paper, we build upon that work and extend the research in the following areas: 1. The simple graph theory based quantities like centrality was replaced with a structure model based state-of-the-art research in graph neural networks (Battaglia et al., 2018). 2. Model parameters and their uncertainties are inferred from the observations by the Bayesian approach for which the prior distributions were proposed. 3. The relation type or its lack was inferred and interpreted. 4. A parametric trend model based on point process (Goulding et al., 2016) and logistic growth (Balakrishnan, 1991) was proposed for the total amounts of patents. The prior and posterior distribution of the parameters are discussed.

To the best of the authors’ knowledge there is no other work relating to the discovery of hidden interactions of AI patent data in EU countries. The same applies to trend analysis and parametric forecasts. The topic discussed mostly in technical reports like (WIPO, 2019) are simple visual plots which are used to express the rapid growth of AI. We noticed that the majority of trend analyses presented in scientific journals are based on year to year percentage growth or simple linear trends estimated from data without uncertainty estimation or thorough statistical analysis for discrete numbers of patents e.g. (Klinger et al., 2018; Tsay & Liu, 2020). In this paper we seek to address this gap by providing a coherent spatial and temporal description of AI patents in terms of the Poisson distribution.

The next section describes methods and data acquisition and processing steps used in the research. “Results” section presents our findings in numerical experiments and “Conclusion” section concludes the paper.

## Materials and methods

Patent information could be obtained from different sources. Starting from publicly available databases like EPO, OECD or Google Patents to the more advanced commercial ones like Derwent, PATSTAT or Global Patent Index. In the following research, we utilized the Global Patent Index database. There is no ’gold standard’ defining which approach is the most appropriate to for retrieving AI patents so different methods are utilised in literature e.g. WIPO approaches were used by Leusin et al. (2020). On the other hand (Fujii & Managi, 2018) followed the practice of the USPTO. We chose the IPO search strategy approaches because, as the authors highlighted, it was deliberately narrowed to ensure that the retrieved patents each have a high presumption of relating to AI.

**Fig. 1:**
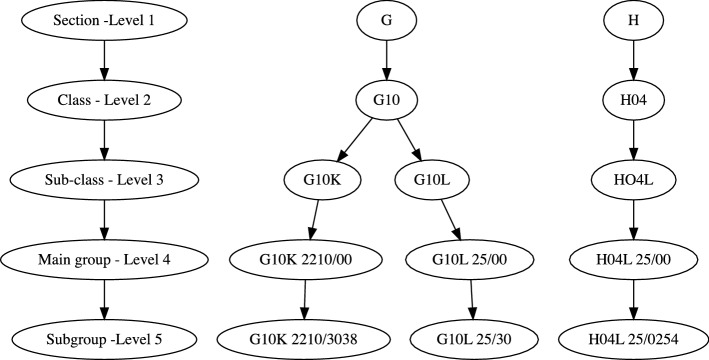
Scheme of hierarchical structure of Cooperative Patent Classification

AI has applications in a broad range of technology areas, yet, patents classified in those technology areas do not necessarily always relate to AI. There is also a wide variety of possibilities for searching patents on a given topic, which includes the use of keywords and the use of patent classification schemes. Patents are classified under either the International Patent Classification (IPC) scheme or the Cooperative Patent Classification (CPC) scheme, according to the technology areas they relate to. The CPC scheme is more detailed than the IPC scheme, but the IPC scheme is more widely adopted worldwide. The CPC is an extension of the IPC and is jointly managed by the EPO and the US Patent and Trademark Office. Hence in our analysis we utilised CPC. Figure 1 presents a hierarchical structure of some exemplary AI patents which could be considered as a tree graph. As a example an interpretation of hierarchical structure of subgroup “H04L25/0254” is as follows:“H”—electricity; “H04”—electric communication technique; “H04L” transmission of digital information, e.g. telegraphic communication; “H04L 25/00”—adapted for orthogonal signalling; “H04L25/0254” using neural network algorithms.

It is not only strategies for identifying AI-related patents that could be ambiguous. There are also different approaches depending on the country affiliation of a specific patent, whether that be the Inventors’ country (INVC) or the Applicants’ country (APPC). Patent counts by Applicants tend to measure the degree of control on patents by each country’s residents, wherever the invention is made. It reflects the innovativeness of firms of a given country, whatever the location of their research facilities. As presented in our research, the Applicants’ country approach simplifies every patent to Applicants (often companies’ headquarters) affiliated to a given country- this skips existing, often, worldwide located subsidiaries. Having said that, we also tested our method on a dataset linked by INVC field like in Fritsch and Wyrwich (2018). Results are in Appendix 3.

### Data description

The research examines the cooperation network in EU countries referred to their code names: CY- Cyprus, MT- Malta, LV- Latvia, LT- Lithuania, EE- Estonia, HR- Croatia, BG- Bulgaria, SK- Slovak Republic, RO- Romania, SI- Slovenia, CZ- Czech Republic, HU- Hungary, PL- Poland, namely the so-called EU-13 and LU- Luxembourg, PT- Portugal, EL- Greece, IE- Ireland, DK- Denmark, ES- Spain, BE- Belgium, FI- Finland, AT-Austria, SE- Sweden, NL- Netherlands, IT- Italy, GB- United Kingdom, FR- France, DE- Germany, collectively named EU-15 (because the period considered was from 1990 the United Kingdom was also included in our analysis despite it having left the EU in 2020). To identify relevant and comparable techniques, we used 76 subgroups (see Level 5 on Fig. 1) of the CPC scheme proposed in the Intellectual Property Office report, see p.32 in IPO (2019), to identify AI. To extract AI patents for EU countries in the time period from 1990 to 2021 (18th of September) we formulate the query in the Global Patent Index (GPI 2021/28) database as detailed in Appendix 1. As a result, we obtained 10,759 AI patents families (a family is a set of all patents in different countries that protect the same invention). As a cooperation indicator, we used *Applicant country of residence* content. The presence of authors from at least 2 different countries indicates cooperation. An example patent having six applicants from [DE, LU, DE, DE, BE, BE] accounts for cooperation between all pairs of 3 countries [LU, DE, BE]. The strength of the cooperation is measured by counting the number of relations. From 10,759 patents families, we identified 1644 (15.3%) patents that have cooperation with at least one additional country—we will refer to them as *cooperation patents*.

The cooperation network was created as a graph with nodes representing countries and edges representing relations. The literature concerning an analysis of AI international collaboration mostly focuses on countries worldwide [e.g. Tang et al. (2022) or Tsay and Liu (2020)]. In our dataset—except for EU countries—we had more than 40 non-EU countries. Since our main goal was to analyze EU countries, to avoid too much dispersion in our analysis, all the non-EU countries are represented as a single entity *Others*. The graph contains an edge only if there was at least one relation—*cooperation patent* (if we use all countries in our dataset the cooperation graph would have almost 70 nodes instead of 28. This, in our opinion, could diminish interpretation of results for our targeted group—EU countries). Patents assigned to a single country are discarded in the relation network—no self-connection is present in the graph (however they contribute to the node feature). Having said that, the information about total patents issued by a given country is retained in node attributes. We utilized fractional counts applied to patents with multiple applicants. When a patent was invented by several inventors from different countries, the respective contributions of each country were considered. This is done to eliminate multiple counting of such patents. For example, a patent co-invented by 2 French, 1 Belgian, and 1 German resident will be counted as 1/2 patent for France; 1/4 for Belgium; and 1/4 patent for Germany. The total (fractional) number of patents is a node attribute in the relation network, while the edge attribute is the total number of relations.

Quantitative analysis of cooperation in patents with relation to various geographical and socio-economical factors was studied e.g. by Tang et al. (2022). In this work, we take a different approach. Instead of explaining relations by exogenous variables, we aim at finding the structure in patent data alone. In particular, we focus on the significance of the level of interactions. This way we simplify the model building process by limiting it to a single dataset and, what’s more important, we avoid the case of missing an important predictor. The structure discovered in this work can be easily enriched with the interpretation obtained from other factors, or due to our Bayesian set up can be added to the model in a form of informative priors.

### Relational regression mixture models

Let $C_{ij}\in {\mathbb {N}}_0$ be the total number of patents shared between countries *i* and *j* (*cooperation patents*). Furthermore the amount of patent of the *i*-th country will be denoted by $C_i\in [0,\infty )$. Following (Battaglia et al., 2018) we propose the relational inductive bias based on a Poisson regression model to relate the amount of patents and the number of interactions:

1 $$\begin{aligned} C_{ij} \sim \text {Pois}\left( \lambda _{ij}=e^\beta (C_iC_j)^\alpha \right) . \end{aligned}$$

Such a model has multiple advantages: (i) The model is simple and easy for interpretation. (ii) Permutation equivariance doesn’t require special ordering. (iii) Setting $\alpha =1$ yields the popular gravity model (without distance term). (iv) The canonical link function of the Poisson distribution is logarithmic so in the $\log$ domain the model can be expressed in its simple linear form:

2 $$\begin{aligned} \log \lambda _{ij}=\alpha \log (C_iC_j) + \beta . \end{aligned}$$

Conditional distribution of $C_{ij}$ is multimodal and classical Poisson regression gives poor results, in particular we observed a heteroskedastic variance of residuals because values much higher than one would be expected from the Poisson distribution. The problem of overdispersion can be overcome by using a mixture of Poisson distributions as a conditional distribution (Lamont et al., 2016). Such a model is known as the regression mixture model (Leisch, 2004).

In this representation every pair of countries *i*, *j* gets a hidden categorical variable $Z_{ij}\in \{0,1,2\}$. Denoting $\lambda _{ijk}={\textsf{E}} (C_{ij}\mid Z_{ij}=k)$ and $x_{ij}=\log (C_i C_j)$ we propose the following model of the relation intensities $\lambda$:

3 $$\begin{aligned} \log \lambda _{ijk} = \alpha _k x_{ij} + \beta _k \quad k\in \{0,1,2\}. \end{aligned}$$

Equation (3) is a system of three equations parameterized by $\alpha _k,\beta _k$ defining three possible behaviours of the cooperation. Since $Z_{ij}$ is an unknown hidden variable we model it by a categorical probability distribution with parameters $\pi _{k}={\textsf{P}}(Z_{ij}=k),\quad \sum _k\pi _k=1$. Hence, the conditional distribution of $C_{ij}$ is given by:

4 $$\begin{aligned} {\textsf{P}}(C_{ij}=c\mid x_{ij})=\sum _k\pi _k p_{\text {Pois}}(\lambda _{ijk},c), \end{aligned}$$

where $p_{\text {Pois}}(\lambda ,\cdot )$ is the probability mass function of the Poisson distribution of rate $\lambda$. Equation (4) can be used to estimate parameters of the model ($\theta =(\alpha _0,\alpha _1,\alpha _2,\beta _0,\beta _1,\beta _2,\pi _0,\pi _1,\pi _2)$) by using the conditional maximum likelihood. Alternatively one can use it to construct a Bayesian posterior distribution of the parameters. Given the parameters $\theta$, the true counts of relational patents $y_{ij}$ and features $x_{ij}$ one can follow (Murphy, 2012) and compute the posterior distribution of the hidden variable $Z_{ij}$ as:

5 $$\begin{aligned} {\textsf{P}}(Z_{ij}=c\mid x_{ij},y_{ij},\theta )=\frac{\pi _c p_{\text {Pois}}(\lambda _{ijc},y_{ij})}{\sum _k\pi _k p_{\text {Pois}}(\lambda _{ijk},y_{ij})}. \end{aligned}$$

Parameters $\theta$ in Eq. (5) are marginalized (averaged) over the posterior distribution. The hidden discrete variable $Z_{ij}$ can be interpreted as the unknown type or class of relation between two countries. Inferring the most probable class, is equivalent to identification of this type. This way the regression model performs also clusterisation, and allows to infer the latent relation type from the patent counts.

### Logistic growth of inhomogeneous Poisson process

Patent application and their publication can occur at any time (to within a granularity of days and working hours). Thus, any aggregation to month or year, results in some information being lost. A natural way of modelling such a phenomenon is a point process (Daley & Vere-Jones, 2006). In particular the Poisson process is a common choice. We follow this approach with the exception that the process cannot be stationary because a huge change in patenting dynamics can be observed over the last decade. A realistic model of the patent publication process is an inhomogeneous Poisson process (Daley & Vere-Jones, 2006). Such a process is an extension of the stationary Poisson process whose rate changes over time.

We argue that patents are similar to other goods thus their production can be described by the logistic curve, commonly used in economics, ecology and other areas (Twiss, 1992; Balakrishnan, 1991). Since patent production is random in nature we propose to model cumulative rate function (6) (expected number of patents produced until time *t*) instead of the cumulative patent counts.

6 $$\begin{aligned} \Lambda (t) = \int _{-\infty }^{t}\lambda (t)\textrm{d}t = \frac{L}{1+e^{-\frac{t-t_0}{s}}} \end{aligned}$$

Such a curve is parameterized by the capacity $L>0$, the midpoint $t_0$ and the scale $s>0$ (an alternative and also commonly used parameterisation involves the rate equal to 1/*s*). The capacity represents the asymptotic maximum at infinity, while the maximum rate is at the midpoint. This is also the point in time where the growth starts slowing down. The scale controls the width of the growth period (10*s* contains almost 99% of the events). Those three parameters describe the dynamics of the process totally and can be estimated from the observations. Let ${\varvec{t}}$ be a vector of *n* random points in time representing patent publication date as a real number. The log likelihood function of those observations is given by Daley and Vere-Jones (2006) as:

7 $$\begin{aligned} \ell ({\varvec{t}}) = \sum _{i=1}^n \log \lambda (t_i)-\int _{t_1}^{t_n}\lambda (t)\textrm{d}t. \end{aligned}$$

One can estimate the unknown parameters $\theta =(L,t_0,s)$ by maximizing (7) or by using $\ell ({\varvec{t}})$ to construct a Bayesian posterior distribution of the parameters. In this paper we followed the approach used for spatial relation discovery and estimated parameters via Bayesian inference. Since the likelihood function involves calculations on thousands of patents, we used approximate yet much faster variational inference (van de Schoot et al., 2021).

The beginning of a new method in Machine Learning (ML) would definitely break the simple logistic growth model. Indeed, in the case of patent data, we observed this in numerical experiments. A single logistic curve doesn’t fit well to the observations. However a superposition of two logistic curves can explain the data with much lower error. In particular we observed a better match of inflexion points as the logistic curve on log scale is asymptotically linear at $t\rightarrow -\infty$ and this is not observed in the dataset. The second curve adds necessary curvature. The final cumulative patent rate can be expressed as:

8 $$\begin{aligned} \Lambda (t) = \sum _{i=1}^2\frac{L_i}{1+e^{-\frac{t-t_{0,i}}{s_i}}}= L\left( \frac{p_1}{1+e^{-\frac{t-t_{0,1}}{s_1}}}+\frac{1-p_1}{1+e^{-\frac{t-t_{0,2}}{s_2}}}\right) . \end{aligned}$$

The capacity of this new rate is $L=L_1+L_2$. By setting $p_1=\frac{L_1}{L_1+L_2}$ and $p_2=\frac{L_2}{L_1+L_2}=1-p_1$ we can observe that (8) corresponds to the cumulative density function of a mixture of logistic distributions (Balakrishnan, 1991) scaled by *L* where $p_1,p_2$ are mixing probabilities. This observation allows us to use numerical procedures for the mixture distribution in a totally different context. Furthermore $\lambda (t)$ corresponds to the scaled probability density function (pdf). This is important for numerical estimation as $\log \lambda (t)$ can be calculated from log pdf which is quite often provided by numerical libraries with an optimized implementation.

### Bayesian inference

To fit the relational model to the graph data we adopt a Bayesian approach. In this way the uncertainty of the model is captured via a posterior distribution of parameters (van de Schoot et al., 2021; Murphy, 2012). This scientific method is commonly used for hierarchical models e.g. (Forthmann & Doebler, 2021), where maximum likelihood methods are not very efficient (Murphy, 2012).

In Bayesian statistics, every parameter is represented not by a single number but rather as a distribution of possible values (van de Schoot et al., 2021). We can distinguish two distributions of parameters: the prior and the posterior. The prior distribution captures our uncertainty about the parameters before obtaining any experimental evidence. It expresses our knowledge or beliefs about the typical values of the unknown parameters. Once the experiment is conducted, the collected data changes the distribution of parameters. This new distribution is called the posterior distribution and it is given by the Bayes theorem.

For Bayesian inference, a joint prior distribution is specified for all the unknown model parameters. Since all the quantities in the relational model are real numbers the prior distribution is assumed to be normal. Furthermore, we assumed a priori parameters independence. The hyperparameters for this distribution are set using a polynomial fit in the log transform domain and widened appropriately to avoid biasing the model towards maximum likelihood estimates. When the experiment is repeated for time series, the Bayesian approach has the advantage that today’s posterior may play the role of tomorrow’s prior.

For temporal analysis, we have less informative priors. In particular, the capacity was modelled using a log-normal distribution with a heavy tail. Midpoint’s priors were weakly informative independent normal distributions and the scale priors were exponential distributions—the least entropy prior. Furthermore, the posterior was estimated using stochastic variational inference with independent normals used as a surrogate posterior (Murphy, 2012).

All the methods used in this research are implemented on top of TensorFlow Probability (Dillon et al., 2017) and within DeepMind JAX ecosystem (Babuschkin et al., 2020). The implementation is available as open-source software.^1^

## Results

As a result of a formulated query (see Appendix 1) into our data set we observed 9 sections, 99 classes, 328 sub-classes, 460 main groups and 8767 subgroups (it is worth noting that single patents could be simultaneously classified as a few sub-classes or main groups or many different subgroups). Analysing only subgroups, classified as AI, the most popular subgroups were: G06N20/00 (computer systems based on specific computational models—Machine learning), G06N 7/005 (computer systems based on specific mathematical models—Probabilistic networks), G05D1/0088 (systems for controlling or regulating non-electric variables—control of position, course or altitude of land, water, air, or space vehicles, e.g. automatic pilot—characterized by the autonomous decision making process, e.g. artificial intelligence, predefined behaviours).

### Spatial relation

**Fig. 2:**
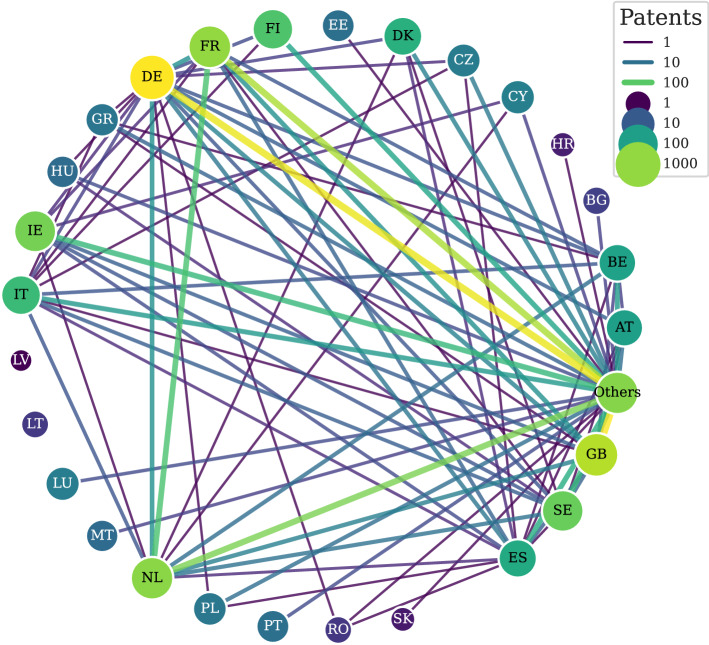
Cooperation network of the AI patents

We represent the cooperation network of the AI patents as a graph where nodes are countries, and edges represent relations based on AI collaboration—see Fig. 2.

The size and colour of nodes represents the total amount of patents ($C_i$) in log scale, similarly the width and colour of edges expresses the number of collaborations also in log scale ($C_{ij}$). As can be observed, the number of AI patents (as a sum over the years 1990–2021) among the EU member countries varies greatly between countries. From the analysis a manifestation of the Pareto principle is observed i.e. most patents are issued by only a few countries. Accordingly, the greatest number of patents was assigned in Germany, followed by the United Kingdom, France and Netherlands. While for Slovenia, Slovakia, Lithuania and Romania there was only a single patent. Results from the cooperation graph shows that in EU countries the greatest number of collaboration patents was with United Kingdom. In total 586 cooperation patents include 509 with non-EU countries and 22 with France–United Kingdom’s top partner in the EU. Germany scored 514 cooperation patents. Out of those 411 were with non-EU countries and only 22 with France—again the top partner in the EU. France itself has 373 cooperation patents. Still, most of them (230) are with non-EU countries, however, France has stronger cooperation within EU and scored 83 patents with the Netherlands—its top partner. At the other end of the scale, a lack of cooperation with other countries was observed in Estonia, Latvia, and Slovenia. For the majority of the countries with cooperation patents most of their partners were non-EU countries. In terms of percentage share with non-EU countries, the data shows the following: Finland—87%, United Kingdom—86%, Germany—80%, Ireland—75% and France—61%. Since “Others” is a meta node representing multiple countries, its cooperation power is expected to be stronger than for a single country. Less obvious is the relation between the number of cooperation patents to the total number of patents for each EU country. Considering the ratio of former and later, the highest share is observed in Bulgaria (37%) followed by Romania ( 20%), Hungary (20%) and Czech Republic (18%)—the countries with a relatively low number of patents. We don’t consider the Slovak Republic because there was only 1.75 patents in total- all with different countries. On the other hand, the lowest percentage shares were observed in Finland (5%), Ireland (4.8%), Luxembourg(4%) and Denmark (4%) (Croatia, Estonia, Latvia and Lithuania don’t have any cooperation patents and in Slovenia there are no AI patents registered).

These basic statistics and the cooperation network (Fig.  2) are definitely informative about the EU patent network yet their interpretation can be misleading. We observe a very high dynamic range of data: some countries have only a few patents while others have hundreds or thousands—hence the use of $\log$ transformation. Without rigorous statistical analysis we have no means of assessing uncertainty in the statistics regarding the relations, especially for the countries at the lower end of patent ranking. Furthermore, the lack of cooperation patents between two countries does not necessarily imply the lack of cooperation between them. There might be simply not enough patents for the relation to be observed.

The numerical results suggest that regression mixture models allow for the inference of both the presence and the strength of patent cooperation between EU countries. Let us consider the network (Fig.  2) as a full graph with with 29 nodes (28 EU countries and “Others”) attributed with the fractional number of patents for a given country. Edges in the graph are labeled by the total number of interaction patents. The graph model gives the distribution of edge features (of total number of cooperation patents) from the node features (fractional total number of patents). We specifically propose to use a mixture of three Poisson distributions as described in “Relational regression mixture models” section. In our Bayesian approach to statistical inference we work with simple, slightly weakly informative priors with one exception for $\alpha _0$ where we propose a deterministic distribution at 0 so $\alpha _0$ de facto is not a parameter and component 0 doesn’t scale with node features.

**Fig. 3:**
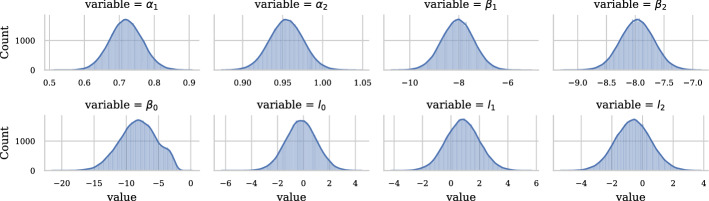
Marginal posterior distributions

For the numerical experiment we propose the following priors for the remaining parameters. The slopes $\alpha _1$ and $\alpha _2$ are normal with mean 0.5 and standard deviation 0.5. This prior allows for both positive and negative slopes with higher probability mass on the positive side. All biases $\beta$ are independent normal distributions with mean -8 and standard deviation 3. Finally, mixing probabilities $\pi _k$ are parameterized in the unconstrained domain by logits $l_k$: $\pi _k=\frac{e^{l_k}}{\sum _je^{l_j}}$ The logits of mixing probabilities are independent centered normals with standard deviation 2. Here we use an overparametrized yet simpler prior where all three logits are specified, and the normalization is encoded in the model. A more detailed discussion of prior selection is presented in Appendix 2 Since the posterior distribution is analytically intractable the inference works with 4000 MCMC (No U-turn sampler) samples from 16 independent chains resulting in 64,000 posterior samples whose marginal distributions are presented in Fig. 3. Numerical values of means and standard deviations are collected in the first two rows of Table 1.

The independent chains allowed us to assess the convergence to the stationary distribution. For all the variables the potential scale reduction (R-hat) is very close to one, so we consider the samples to come from the stationary distribution.

**Table 1:** Marginal moments of posterior distribution

	$\alpha _1$	$\alpha _2$	$\beta _0$	$\beta _1$	$\beta _2$	$l_0$	$l_1$	$l_2$
Mean	0.719	0.955	− 8.146	− 8.044	− 7.966	− 0.186	0.818	− 0.613
Std	0.046	0.021	2.873	0.648	0.291	1.193	1.173	1.166
2020								
Mean	0.726	0.906	− 12.099	− 7.910	− 7.147	− 0.225	0.951	− 0.579
Std	0.020	0.010	2.834	0.275	0.140	0.510	0.473	0.463
2019								
Mean	0.745	0.909	− 12.031	− 7.863	− 6.962	− 0.396	0.994	− 0.491
Std	0.032	0.017	2.909	0.424	0.217	0.803	0.745	0.738
2018								
Mean	0.766	0.911	− 11.990	− 7.859	− 6.744	− 0.734	1.142	− 0.360
Std	0.058	0.030	2.996	0.742	0.382	1.328	1.215	1.206

**Fig. 4:**
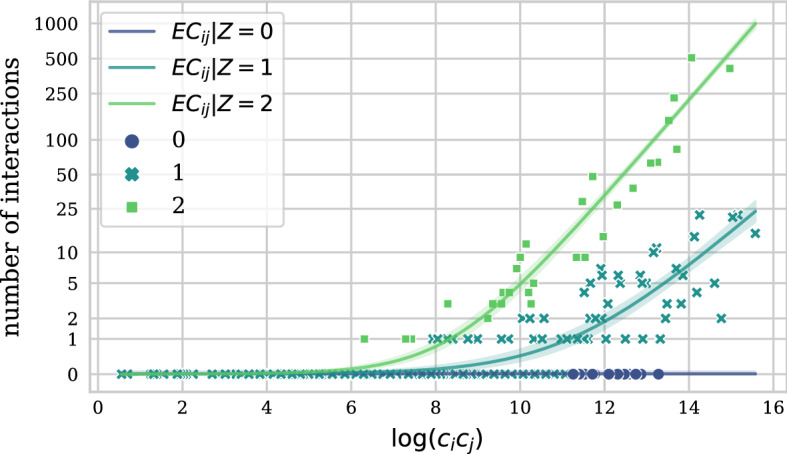
Relational mixture regression with the inferred relation type

Each posterior sample represents a single relational model consisting of three Poisson regression models being the components of the mixture. Their collective behaviour is presented in Fig. 4, where the mean of each component is visualized independently. The confidence ribbons show 95% credible intervals for the means obtained from the posterior samples as empirical quantiles.

By design, the means of the last two components depend on nodes features, while the first one is independent of the features ($\alpha _0=0$). Since slope (exponent) describes how fast the average number of cooperation patents grows with nodes features we argue that its value quantifies the strength of the relation. Both slopes ($\alpha _1,\alpha _2$) are positive and the posterior probability of having negative slope is ${\textsf{P}}(\alpha _i<0)<0.00025$. This means that the increase in total patents yields an increase in cooperation patents as well (rate is an increasing function of features). The third component has $\alpha$ almost one and this relation corresponds to cooperation patents with “Others”. Interestingly this relation has almost the form of the gravitational model. The within-EU cooperation is mostly covered by the second component. However, the most interesting is the first component- the one whose slope was a priori set to 0. Hence, its average does not depend on node features, and we associate this component with the lack of long-lasting cooperation between given countries. The reasoning is that if there were cooperation, an increase of total patents would increase the number of cooperation patents. The statistical model allows us to infer which pairs of countries do not show any evidence of cooperation. There are a high amount of patents fitting this description, yet their cooperation is weaker than between other countries of similar patent statistics.

**Fig. 5:**
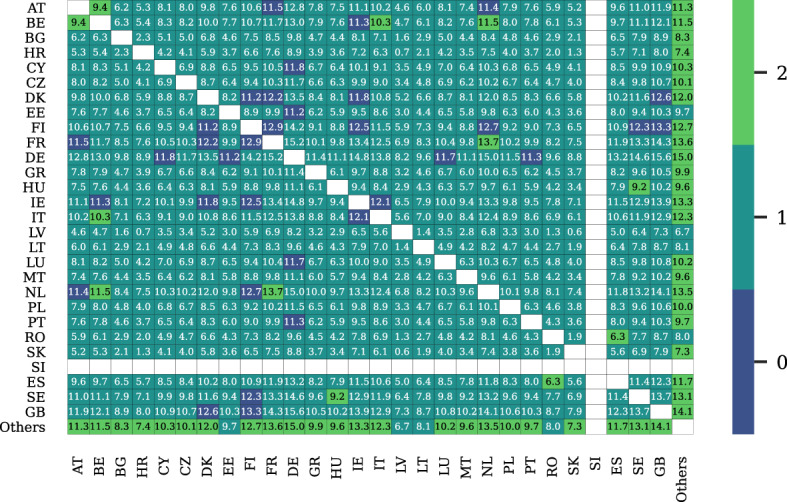
The most probable type of interaction between countries labeled with $\log$ edge features. Type 0:FR-AT, NL- AT, IE-BE, DE-CY, FI- DK, FR-DK, GB-DK, DE-EE, FR-FI, IE-FI, NL-FI, SE-FI, GB-FI, LU-DE, PT-DE, DK-IE, IT-IE. Type 2: mainly concerned EU countries with non-EU countries (Others), as well as within the EU between AT-BE, BE-IT, BE-NL, RO -ES, FR-NL, HU-SE

Given the model and its parameters, every pair of node and edge features can be assigned a distribution of the hidden variable $Z_{ij}$. This way it is possible to estimate the most probable type of relation between two countries. This clusterisation is visualized in Fig. 5.

For 28 EU countries and “Others”,there are 406 possible connection combinations (everyone with everyone). The results showed that 17 pairs of countries have low cooperation (Type 0); a cooperation level of 332 was average (Type 1) and in 29 cases the cooperation was high (Type 2) (Slovenia was removed because of no patents). As previously mentioned, type-2 relations (high cooperation) mainly involved EU countries with non-EU countries (“Others”), as well as within the EU between AT-BE, BE-IT,BE -NL, RO -ES, FR-NL, HU-SE.

Type-1 represents most of the pairs with cooperation patents plus some pairs without cooperation patents. In this case we argue the total amount of patents is not large enough, and the resulting rate is so low, that a zero interactions result has high probability. On the other hand, the lack of cooperation patents between countries with high numbers of total patents is an indication of a type-0 relation. This interpretation is consistent with the results from the models at different times.

In Table 1 we provide additional statistics of parameters based on time-limited subsets of the main dataset. For example, the data in the rows for 2018, show parameters for the posterior distribution for patents published by the end of 2018. Priors were mostly the same except for $\beta _0$, where we were forced to use a more informative prior of average − 10 due to greater noise in the observations. Priors for the subsequent years were just posteriors from the previous years. The results are stable and estimated posterior distributions are quite similar over the years—this shows our current estimates can be useful for further analysis in the future.

Furthermore, the posteriors are also close to the main posteriors estimated for the whole dataset. A minimal increase of uncertainty can be observed for all $\alpha$ this is probably because of less informative priors and noisy observations from the pandemic year 2021. The value of $\beta _0$ seems to be highly determined by the choice of the prior distribution and it makes sense as the only evidence for it is the lack of cooperation patents. Having said that, the case of no cooperation patents still can be grouped into their types. Furthermore, over the 3 years as the number of patents grow we observed an increase in the number of type-0 relations. As expected, the additional observations improve the discovery of a significant lack of cooperation.

The Bayesian analysis of interactions according to the rule that today’s posterior is tomorrow’s prior suggests that the proposed model holds over time and the only change we can expect is the discovery of more non-interacting pairs of countries.

Numerical results gave us a clear structure of the patenting network. Some parts of this structure could be explained by external factors deliberately omitted from the model e.g. information about relation with *Others* can explain most of the Type-2 relation.

Analyzing literature concerning international collaboration e.g. (Acosta et al., 2011) or (Fritsch & Wyrwich, 2018), also suggests that factors like economic or geographical distance may influence the type of relation. It is also worth noting that EU countries vary from each other in many aspects like digitalization, economic conditions, industry, investment in R &D development, number of ICT companies, level of innovativeness, number of international partnerships, and many others. All these aspects could affect international cooperation. However, in this paper, we focus on exploring the existence of such patterns. Detailed analysis of international patent cooperation of AI and the other factors goes beyond the scope of this article. Further analysis e.g. regression models (Tang et al., 2022) could provide more intuitive insight into the inferred structure.

### Temporal dynamics

Analysing the total number of AI patents over the period 1990–2021 from our data set, we could observe that the growth rate was very dynamic over the last 5 years. However, from a simple quantitative assessment, we can’t infer a trend and expected future changes. Although in the literature it is hard to find reliable analysis based on statistical methods. Majority trends in scientific publications and reports are based on percentage growth. These are simple trends estimated from data without model regularization, or uncertainty based on the quantitative patent data or analysing trends in most prominent areas in AI. However, in periods, when many companies are investing in AI development, (including investment in infrastructure, data and staff training) it is important to have a long-term perspective on those technologies.

Patent information is a valuable resource for assessing technology trends. In the history of AI, few technology booms were observed. The previous section described spatial relations between countries, however, the patent landscape is dynamic and changes rapidly over time. In this section, we discuss the temporal behaviour of the results. Since modelling interactions between 29 elements over time would require a complicated spatio-temporal model, here we focus on finding the global trends in AI patents.

As the patent can be published at any time we use the inhomogeneous Poisson process to describe this process. Furthermore, we propose the use of a logistic curve to model the patent production process. Such a model is simple and more technically correct for point data (patent counts are integer numbers—they must be modelled by a discrete probability distribution) compared to simple trend lines estimated by the least-squares method. In particular the classical regression is based on normal distribution of residuals. Such a distribution can be inaccurate for discrete data and may result in wrong estimates of the uncertainty. However, because patent counts are integer numbers they must be modelled by a discrete probability distribution.

Also, no aggregation means less noise in the data. This is a common issue of methods based on aggregation (monthly or yearly), where the last period is incomplete and usually discarded from the analysis. With the point process approach, we overcome this problem because the model is trained on time differences between successive events.

**Fig. 6:**
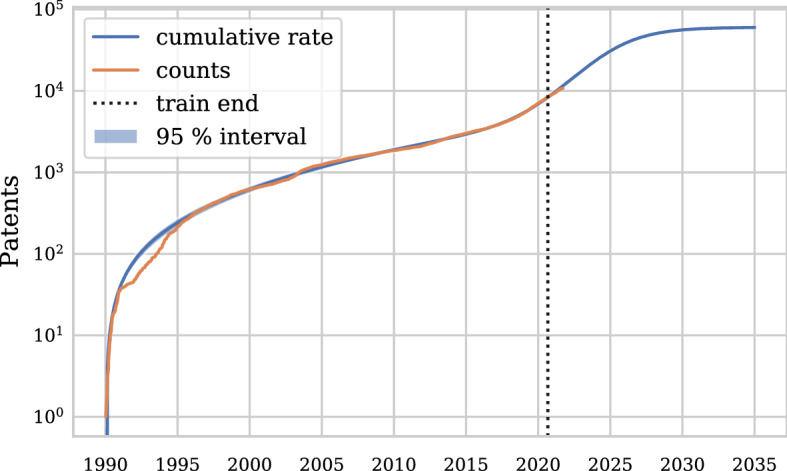
Cumulative rate

As in the case of spatial relations, we use a Bayesian posterior distribution to capture the uncertainty of the forecasts. For the numerical experiment, we propose the following priors. The Pareto prior for capacity *L* is parameterized with concentration 1.1 and scale 11,000 to give an average of 50,000 and infinite variance. The weakly informative prior was selected such that most of the probability is after 2021 as *L* is the asymptotic number of patents at infinity. Midpoints $t_0$ are independent normal distributions with their mean in January 2011 and with standard deviation 27 years(which results in almost a century above the mean for probable locations of the midpoints by 6 sigma rule). The scale parameters are independent exponentials (Maximum entropy prior) with an average of 137 years (50,000 days). Finally, the fraction parameter $p_1$ (specified as logit $l_1$) is normal of unit average and standard deviation. Since we used, variational inference (van de Schoot et al., 2021; Murphy, 2012) to approximate Bayesian inference. The moments of posterior distributions (mean-field- an independent normal approximation of the true posterior) are collected in Table 2. The resulting trend is visualized in Fig. 6.

**Fig. 7:**
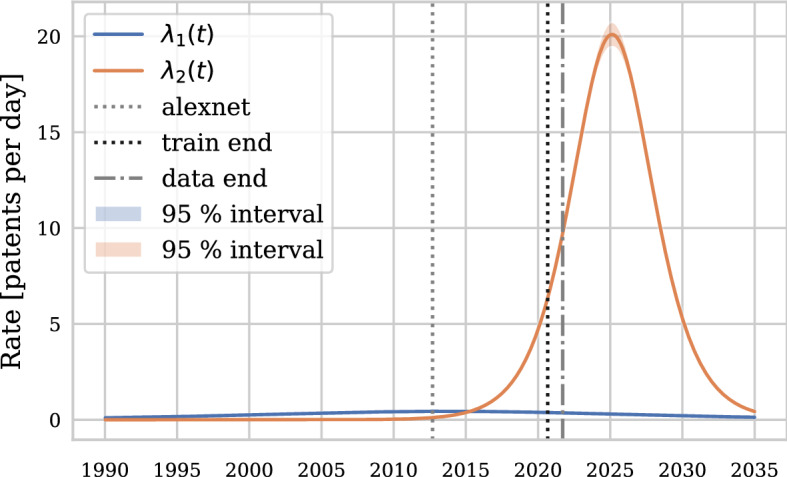
Rate components

**Table 2:** Posterior moments of trend model

	$t_{0,1}$	$t_{0,2}$	$s_{1}$	$s_{2}$	$p_{1}$	*L*
Mean	2013-10-02	2025-02-15	3197 days	690 days	0.092	61,215
Std	182 days	23 days	140 days	8 days	0.002	659

The trend was estimated on all patents published before ’2020-09-01’ and the last year of observation was used for model evaluation during the training. The training involved maximization of the evidence lower bound (ELBO) if we observed an increase of the expected likelihood on the validation patents (after 2020-09-01). Both the expected likelihood and Kullback–Leibler divergence were estimated by the Monte Carlo method with 16,384 samples from the surrogate posterior.

The estimated trend (together with its 95% credible interval) is compared to the cumulative number of patents up to a given time. Except for the period in the early 90’s we observe high accuracy of the trend line. This trend line extrapolates patenting cumulative rate in the future until 2035. We can expect the cumulative rate to begin saturating after 2025. Further analysis is difficult from such a plot and the detailed rate functions depicted in Fig. 7 reveal the forecasting behaviour. The rates correspond to the two components of the logistic growth curves we used in the model. Given the times and the behaviour of the rate, we can speculate about their nature. We can observe that $\lambda _2$ took off after 2012—a time we consider as the beginning of the deep learning era, see the dotted line (alexnet) marking the publication of Krizhevsky et al. (2012). This suggests that $\lambda _1$ must correspond to the remaining AI technologies—shallow learning. The rate of this part of patents has already begun slowing down around 2013/2014. We cannot be more precise as the credible interval for the corresponding midpoint (see $t_{0,1}$ in Table 2) is over 2 years wide (± two standard deviations). Our trend predicts that deep learning will also begin slowing down around the first quarter of 2025 after reaching the peak rate of over 20 patents per day. The credible interval, in this case, is much narrower (of the order of months). The estimated capacity is above 61,000 patents so the current number of patents is expected to increase 6 times. Over 91% (1-$p_1$) of them can be attributed to the deep learning that will last around 11 years from its beginning. The shallow learning part will last a lot longer and its overall time span can be estimated to be 52 years. This again shows a Pareto rule: 91% of all patents are produced during the period spanning only 21% of the AI time.

## Conclusion

AI technologies are currently one of the most rapidly developing areas of ICT, which could be easily used in all areas of technology, solving many problems in healthcare, automotive, industry and many others. AI is expected to play a major role in shaping global competitiveness and productivity over the next decades. However, Europe lags in AI-related patent applications compared with e.g. the United States. When it comes to investment, the United States and China are also ahead of Europe. There may be more than one reason for this e.g. Europe’s private research, development, and innovation investments are lagging compared to China, the United States, Japan or South Korea. Hence EU policymakers need to encourage this strategic technology through dedicated programs, initiatives and legislation. Fortunately, policymakers at the EU and national levels appear to recognize the importance of AI which is reflected in some EU AI strategies. Nevertheless, it is worth highlighting that much more has to be done.

To achieve EU competitiveness in AI there are a host of steps that could be done e.g. strengthen its digital single market, attract top global talent, promote a Europe-wide AI ecosystem and collaboration, create dedicated investment funds which include both the public and private sectors, strengthening EU-UK cooperation (because London is Europe’s most important AI hub) and with other non-EU leading countries (Brattberg et al., 2020).

Continued advancement in AI requires collaboration between industry, academia, and government as well as the industry wide development of solutions. So there is an important role for founding research and development programs which incorporate the public and private sectors and could provide a strategic advantage for the EU.

All the decisions and policies can be more efficient if they are data driven. This article analyzes AI patents produced in the years 1990–2021 in the EU countries using the EPO patent database and provides some insights into cooperation and the trends in AI patents. This may boost patenting given the importance of cooperation for innovation.

It is not a surprise that the strongest economies in the EU have produced most of the AI patents. The country with the highest number was Germany, followed by United Kingdom, France, the Netherlands, Italy, Sweden and Finland. The numbers also support the intuition that the countries having a large number of patents will most likely have a large number of common patents. In particular, the number of cooperation patents depends on the product of patents of individual countries by a power law. When non-EU countries are considered as a single meta country “Others” we can identify significantly different types of relationships with “Others”. With regard to most EU countries, it can be generalized that a significant amount of patent cooperation on AI took place with countries outside the EU.

The proposed Bayesian model additionally enables the potential to distinguish between types of interactions between different countries, ordered by the strength of these relations. The key insight from the paper is that the absence of common patents doesn’t imply that there is no cooperation between countries especially those with a low total number of patents. Bayesian inference allows us to distinguish lack of cooperation versus the alternative of low cooperation where the probability of interaction patent is low but not zero. We showed that 17 pairs of countries had no cooperation and the results hold for 4 years of analysis. In those cases, policymakers can try to improve cooperation by launching dedicated research initiatives involving countries with weak cooperation levels. Alternatively—depending on the policy—founding agencies may try to use existing cooperation to network to improve the patent yield from limited science founds.

As time goes by and the total number of patents grows, more pairs are assigned to the none cooperative group. It’s worth noting that future studies can become our posterior distributions according to the principle that today’s posterior is tomorrow’s prior. Having said that we point out that the relationship model is static and does not account for the dynamics of change.

When it comes to temporal dynamics the trend in AI patent application over three decades can be accurately explained by the sum of two logistic curves. By utilising the historical analysis of machine learning publications, the two components of the trend were associated with two trends in the field. The first is a family of classic algorithms used mainly in the 1990s, the second corresponds to deep learning methods. Since the emergence of the growing trend of deep learning, the trend of traditional methods has also been gradually diminishing. We suspect that further detailed analysis of patents categorization would confirm our interpretation. One of the curves has already reached its peak rate in the 2010s and the second one will begin slowing down in the mid-2020s. Having said that the total curve has many decades till saturation.

We would like to emphasize that such a forecast can be biased by the pandemic years 2020–2021 so could be seen as a limitation of our model. Having said that the model could help to assess what would have happened to AI patents had the pandemic not happened. It is worth noting that the international collaboration in AI has accelerated the fight against COVID-19.

Another limitation is that we ignore the possibility that another technology (e.g. efficient probabilistic modelling) could emerge just like deep learning did and start a new growth curve. Historical research on AI is characterized by cycles of growth and decline over several years. The emergence of new technologies causes a sharp increase in interest in the new topic; therefore, the emergence of a new method may result in a further increase in AI patents based on the new technology, beyond the prediction from our developed model. Such a paradigm shift is impossible to predict without strong assumptions about breakthrough occurrence in time.

Having said that we point out that our predictions can be useful for ML researchers to identify emerging trends and find potential partners from the industry. The analysis of patent data itself can stimulate applications of new probabilistic or graphical models, given the rich relational structure between countries, patents, and CPCs.

One aspect where the model could be improved is the forecast uncertainty. Since in its current form the trend model does not account for increased uncertainty of the long term forecast, the credible intervals of the forecast are expected to be too narrow. Future work could solve this issue by modelling logistic growth using a differential equation with additional random components e.g. Wiener process. Moreover despite the fact that we have limited our analysis to the AI patents in EU countries to find a cooperation pattern between EU members, the analysis framework proposed in our paper could be extended to examine observed type-2 relations (high cooperation) which mainly concerned EU with non-EU countries (*Others*). Analysing non-EU countries separately could be a valuable topic for another analysis e.g. to investigate the EU with non-EU countries’ cooperation pattern and with which non-EU countries and EU-member states have the most effective collaboration.

## Figures and Tables

**Fig. 8:**
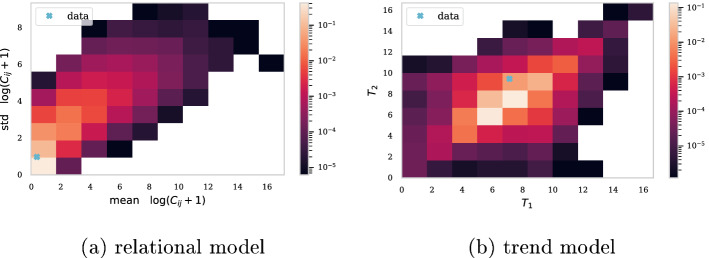
Prior predictive checking

**Fig. 9:**
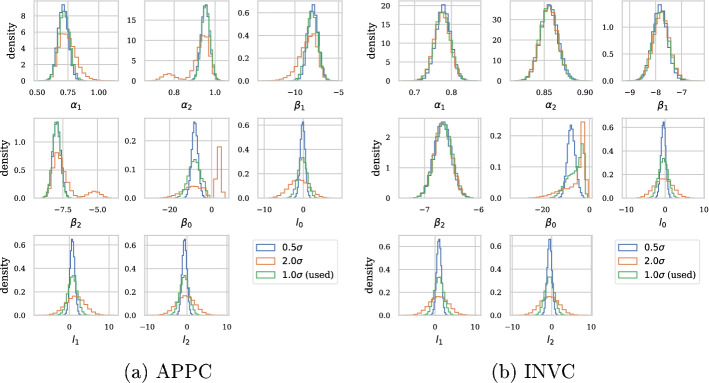
Posterior distributions for different priors

**Fig. 10:**
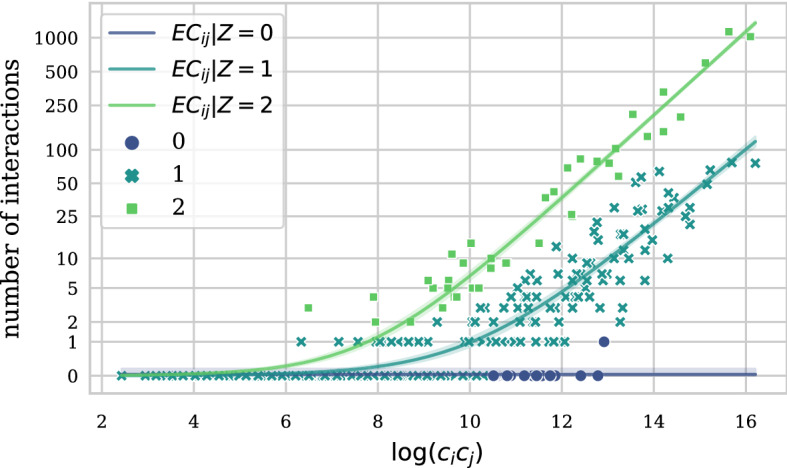
Relational mixture regression with the inferred relation type for model with inventor linking

## Appendix 2: Prior

Prior predictive checking with test statistics being mean and standard deviation of shared patent counts $C_{ij}$ evaluated on 100,000 samples. For trend model we use the number of patents before and after first of January 2005 evaluated on 300,000 samples (Fig. 8).

Additionally, we investigate the proposed prior for the relation network by checking the posterior distribution obtained from the scaled prior. By scaled prior we mean the prior distribution with scale factor scaled by 0.5 and 2.0, which results in twice narrower and wider distributions. Results are collected in Fig. 9a.

A wide prior allows for a multimodal posterior of $\beta _0$. We argue that the choice of prior for this parameter controls the boundary of medium vs low cooperation as high value of $\beta _0$ leads to more pairs are assigned to component 0. We can also observed that under this the wide prior, there is a chance that $\alpha _1$ and $\alpha _2$ as similar in marginal distributions. This is partially due to correlation in the posterior all samples with $\alpha _2<0.8$ correspond to $\beta _0>0$. Given the observation that $\alpha _2$ corresponds to cooperation with *Others* we expect its nature to be different thus we opted for a prior straightening for this difference. Besides the additional modes, the posteriors in general are similar because a wider prior results in wider posterior.

## Appendix 3: Inventor’s country (INVC) approach

In this section, we provide results for an alternative analysis of the cooperation graph based on the inventor country field in the patent database following the approach of Fritsch and Wyrwich (2018) and Fritsch et al. (2019). In particular, Table 3 contains model parameters estimated with the same priors as in the main result of the paper. The results are quite similar, which suggests that the analytical model is quite universal and there may be a mechanistic explanation for such a phenomenon. The similarity is not because of a narrow prior because we verified this with a different distribution reported in Fig. 10.

The posterior samples also yield three types of behavior in the cooperation network as visualized in Fig. 9b. Yet again, the component with the highest $\alpha$ corresponds to cooperation with others. One additional comment regarding INVC linking is that we observe higher dispersion around the mean which may require a distribution of higher dispersion than for Poisson.

**Table 3:** Marginal moments of posterior distribution for model with inventor linking

	Variable	$\alpha _1$	$\alpha _2$	$\beta _0$	$\beta _1$	$\beta _2$	$l_0$	$l_1$	$l_2$
Value	Mean	0.776	0.856	− 5.950	− 7.797	− 6.657	− 0.424	0.971	− 0.563
	Std	0.021	0.011	3.199	0.306	0.160	1.177	1.169	1.167

## Footnotes

1 https://github.com/krzysztofrusek/patents_in_ai.
